# Sodium vanadate combined with l-ascorbic acid delays disease progression, enhances motor performance, and ameliorates muscle atrophy and weakness in mice with spinal muscular atrophy

**DOI:** 10.1186/1741-7015-11-38

**Published:** 2013-02-14

**Authors:** Huei-Chun Liu, Chen-Hung Ting, Hsin-Lan Wen, Li-Kai Tsai, Hsiu-Mei Hsieh-Li, Hung Li, Sue Lin-Chao

**Affiliations:** 1 Institute of Molecular Biology, Academia Sinica, Taipei 115, Taiwan; 2 Graduate Institute of Life Science, National Defense Medical Center, Taipei 114, Taiwan; 3 Department of Neurology, National Taiwan University Hospital and National Taiwan University College of Medicine, Taipei 100, Taiwan; 4 Department of Life Science, National Taiwan Normal University, Taipei 116, Taiwan

**Keywords:** L-ascorbic acid, combined treatment, SMA, vanadate.

## Abstract

**Background:**

Proximal spinal muscular atrophy (SMA), a neurodegenerative disorder that causes infant mortality, has no effective treatment. Sodium vanadate has shown potential for the treatment of SMA; however, vanadate-induced toxicity *in vivo *remains an obstacle for its clinical application. We evaluated the therapeutic potential of sodium vanadate combined with a vanadium detoxification agent, L-ascorbic acid, in a SMA mouse model.

**Methods:**

Sodium vanadate (200 μM), L-ascorbic acid (400 μM), or sodium vanadate combined with L-ascorbic acid (combined treatment) were applied to motor neuron-like NSC34 cells and fibroblasts derived from a healthy donor and a type II SMA patient to evaluate the cellular viability and the efficacy of each treatment *in vitro*. For the *in vivo *studies, sodium vanadate (20 mg/kg once daily) and L-ascorbic acid (40 mg/kg once daily) alone or in combination were orally administered daily on postnatal days 1 to 30. Motor performance, pathological studies, and the effects of each treatment (vehicle, L-ascorbic acid, sodium vanadate, and combined treatment) were assessed and compared on postnatal days (PNDs) 30 and 90. The Kaplan-Meier method was used to evaluate the survival rate, with *P *< 0.05 indicating significance. For other studies, one-way analysis of variance (ANOVA) and Student's t test for paired variables were used to measure significant differences (*P *< 0.05) between values.

**Results:**

Combined treatment protected cells against vanadate-induced cell death with decreasing B cell lymphoma 2-associated X protein (Bax) levels. A month of combined treatment in mice with late-onset SMA beginning on postnatal day 1 delayed disease progression, improved motor performance in adulthood, enhanced survival motor neuron (SMN) levels and motor neuron numbers, reduced muscle atrophy, and decreased Bax levels in the spinal cord. Most importantly, combined treatment preserved hepatic and renal function and substantially decreased vanadium accumulation in these organs.

**Conclusions:**

Combined treatment beginning at birth and continuing for 1 month conferred protection against neuromuscular damage in mice with milder types of SMA. Further, these mice exhibited enhanced motor performance in adulthood. Therefore, combined treatment could present a feasible treatment option for patients with late-onset SMA.

## Background

Spinal muscular atrophy (SMA) is an inherited neurodegenerative disease characterized by motor neuron degeneration in the anterior horn of the spinal cord that leads to muscle atrophy and paralysis [1]. SMA is classified into different types based on the age at onset and disease severity. Symptoms of type I SMA manifest before 6 months of age, and patients never achieve the ability to sit. The onset of type II SMA occurs between 6 and 18 months, and patients are never able to stand or walk. Patients with type III SMA present with symptoms after 18 months, and they are able to walk at some point [2-4]. Two survival motor neuron (*SMN*) genes on chromosome 5q13 have been correlated with SMA: telomeric *SMN1 *and centromeric *SMN2*. SMA is caused by deletions or loss-of-function mutations in *SMN1 *with the retention of *SMN2 *[5-8], resulting in production of insufficient full-length SMN transcripts. *SMN2 *primarily transcribes exon 7-excluded mRNA because of a C-to-T transition at position 6 in exon 7 [9,10] and produces an unstable C-terminally truncated SMN protein. However, patients with SMA present with varying degrees of severity depending on the number of *SMN2 *copies, a finding that has also been replicated in SMA mouse models [7,11,12], indicating that *SMN2 *could serve as the SMA modifier and is therefore a natural target for SMA therapy [12-16].

Two SMA therapy strategies that target *SMN2 *to produce more SMN have been investigated: enhancing *SMN2 *promoter activity and correcting *SMN2 *alternative splicing. Some compounds have been demonstrated to activate the *SMN2 *promoter and/or to change the *SMN2 *alternative splicing pattern, including histone deacetylase inhibitors (sodium butyrate, valproic acid (VPA), trichostatin A, suberoylanilide hydroxamic acid, and LBH589), prolactin, salbutamol, and sodium vanadate (SV) [17-24]. Synthesized antisense oligonucleotides (ASO) have also been shown to effectively reverse the *SMN2 *splicing pattern *in vitro *and *in vivo*, and they have displayed promising efficacy in treating SMA [25-28]. However, many of these compounds are known to be toxic at high doses, and their biosafety for human clinical trials remains to be proven [29,30].

SV is a candidate compound for SMA therapy *in vitro *[23,31]. SV and SV derivatives have been effective in treating diabetes in rodent models [32-34] and are currently in phase II clinical trials [35]. However, high doses or long-term administration of vanadium damages organs and causes reproductive and developmental problems in animals [36-38]. Chelation therapy that combines vanadium compounds with chelating agents capable of binding vanadium *in vivo *to reduce poisoning has been one approach to reducing vanadium toxicity [39-41]. L-ascorbic acid (L-AA; vitamin C) is a natural vanadium detoxification agent that has been demonstrated to be safe for human use [40,42,43]. The interaction between L-AA and SV occurs under physiological conditions and is known to decrease vanadium toxicity [44,45].

In the present work, the therapeutic potential of SV in combination with L-AA (combined treatment) was investigated in a mouse model of late-onset SMA that was previously used as a preclinical therapeutic testing system for SMA [26,46]. The results indicate that combining L-AA with SV does not disrupt the ability of SV to increase the production of SMN levels but it eliminates SV-induced cytotoxicity *in vitro*. Mice with late-onset SMA that received combined treatment on postnatal days (PNDs) 1 to 30 exhibited delayed disease progression and enhanced motor activity in adulthood (PND 90). We also found sustained and elevated SMN levels, increased motor neuron numbers, improved muscle pathology, and reduced Bax levels in the spinal cords of the adult mice. Importantly, vanadium accumulation in the kidneys and livers of these mice was largely reduced, and those organs retained normal function during development and adulthood. Therefore, our study provides a potentially feasible and effective approach to treating patients with late-onset SMA.

## Methods

### Cell culture and chemical treatment

The procedures for culturing NSC34 cells stably expressing *SMN2 *(*SMN2*-NSC34) have been described previously [31]. A dermal biopsy obtained from a patient with type II SMA (a 39-year-old woman with three copies of *SMN2*) was acquired from the Department of Neurology, National Taiwan University Hospital, and primary human dermal fibroblasts (HDFs) were cultured following standard procedures [47]. The protocol for the human study was approved by the Research Ethics Committee of the National Taiwan University Hospital (NTUH-REC no. 201011059RB). Control wild-type (WT) primary HDFs (from a 29-year-old woman) were purchased from Cell Application Inc. (San Diego, CA, USA). Primary HDFs were cultured in fibroblast growth medium (Cell Application Inc.) at 37°C with 5% CO_2 _in a humidified incubator. The cells were plated 1 day before treatment with 400 μM L-AA (Sigma Aldrich, St Louis, MO, USA), 200 μM SV (Sigma), or 400 μM L-AA and 200 μM SV and harvested at the indicated times.

### Cell viability assay

At 1 day before treatment and harvesting, 5 × 10^5 ^cells were plated onto six-well culture plates. Following treatment the total cell numbers were measured by the trypan blue exclusion test using the Countess Automated Cell Counter (Invitrogen, Carlsbad, CA, USA). Cell viability was evaluated three times for each condition.

### Animal models

SMA-like mice were previously generated via a homozygous knockout of *Smn *exon 7 with a transgene of human *SMN2 *(Smn^-/-^SMN2^+/-^) by our laboratory [16]. The mice model of late-onset SMA (Smn^-/-^SMN2^+/+^) used in these studies had four copies of SMN2 and mice were generated via an initial breeding with a mouse model of late-onset SMA [48]. Genotyping was performed as described previously [30]. SMA mice were maintained on a 12-h light and 12-h dark schedule in accordance with the principles of laboratory animal care. The mice were supplied with sterile water *ad libitum *and rodent pellets under the control of the animal facility of the Institute of Molecular Biology, Academia Sinica, Taiwan. All procedures were approved by the Academia Sinica Animal Care and Use Committee, Master Protocol no. RMiIMBLH2008024.

### Drug administration

SV and L-AA were dissolved in sterile deionized water. Vehicle (water), SV (20 mg/kg once daily), L-AA (40 mg/kg once daily), or SV (20 mg/kg) combined with L-AA (40 mg/kg) were orally administered on PNDs 1 to 30 using a 24-gauge feeding needle as described previously [49].

### Western blotting

Cell or tissue lysates (20 μg) were prepared for western blot studies as described previously [31]. Primary antibodies used for western blotting included mouse anti-SMN (1:5,000; BD Biosciences, San Diego, CA, USA), mouse anti-β-actin (1:10,000; Sigma), rabbit anti-Bax (1:1,000; Millipore, Temecula, CA, USA), and rabbit anti-caspase 3 (1:1,000; Cell Signaling, Temecula, CA, USA). Secondary antibodies conjugated with horseradish peroxidase (Millipore) were used at a dilution of 1:5,000.

### Ear morphology analysis

Mice were examined from PND 50 to PND 96 and their ear integrity was determined. The severity of ear morphology was assigned a score from 0 to 4, with 0 indicating the most severe loss of integrity. A score of 4 indicated normal ear morphology, 3 indicated a red color at the tip, 2 indicated purple and black colorations indicative of necrosis, and 1 indicated loss of half of the external ear. A score of 0 indicated loss of nearly all of the external ear. Scores were plotted and analysis of variance (ANOVA) was applied to determine significance.

### Motor activity

Motor functions in the mice were analyzed by a battery of behavioral tests. In the surface-righting assay, each pup was placed in a supine position, and the time to turn over was measured (maximum 30 s) every day on PNDs 1 to 12 [50]. In the geotaxis assay, each mouse was placed on a 30° incline with its head facing the bottom of the incline. Success was judged if the mouse was able to reorient itself 180° within 30 s [50]; measurements were taken daily on PNDs 1 to 12. Hind limb strength was determined by the tube test. Measurements were taken as a mouse hung by its hind limbs on the lip of a 50-ml tube [51]. The tube test was performed daily on PNDs 1 to 12. Locomotion and exploratory movement were measured in the adult mice by the open-field test. Each mouse was placed alone in the corner of an open-field cage (480 × 480 mm^2^) made of polyvinyl chloride. The activity of the animal in the open-field cage was detected for 60 minutes using a video imaging system [52]. Motor function was also measured by the accelerating rotarod test. Each mouse was evaluated as the rotation speed increased from 4 to 40 rpm over 5 minutes; the final score was an average of three trials [53].

### Pathological studies

Mice (n = 3 in each group) were perfused with 4% paraformaldehyde (PFA). Lumbar spinal cords and tibialis anterior (TA) muscles were excised, fixed overnight, dehydrated using an infiltration machine (Leica Microsystems Nussloch GmbH, Nussloch, Germany), and embedded in paraffin. Lumbar spinal cords (4 μm) were serially sectioned at 50 μm intervals, and muscles (4 μm) were cross-sectioned at the midpoint. The sections were mounted on slides and stained with hematoxylin and eosin (H&E). Images were observed using a Zeiss Axio Observer Z1 microscope (Carl Zeiss, Jena, Germany) with a 10 × objective and analyzed using MetaMorph software (v.7.7.2; Molecular Devices, Sunnyvale, CA, USA). Using this software TA muscle area (> 500 myofibers per mouse) and the mean motor neuron numbers per section (> 10 sections per mouse) were determined. For whole-mount immunostaining, TA muscles were excised and fixed in 4% PFA overnight at 4°C. After three washes in 0.1 M phosphate-buffered saline (PBS)/0.1 M glycine, the muscles were blocked in blocking buffer (3% bovine serum albumin (BSA) and 0.5% Triton-X 100 in 0.1 M PBS), followed by incubation with anti-neurofilament H antibody (diluted to 1:500; Millipore) in blocking solution overnight at 4°C. The following day, the samples were washed in five changes of rinse solution (1% BSA and 0.5% Triton-X 100 in 0.1 M PBS) over a period of 5 h and then incubated with Alexa Fluor 488 donkey anti-rabbit IgG and tetramethylrhodamine-5-(and 6)-isothiocyanate (TRITC)-conjugated α-bungarotoxin (α-BTX) (diluted to 1:1,000; Molecular Probes, Carlsbad, CA, USA). After washing with five changes of rinse solution over a period of 5 h, muscle samples were mounted onto slides using fluorescence mounting solution (Invitrogen). Confocal images were captured using an LSM710 microscope (Carl Zeiss) with a 25 × objective lens. Neuromuscular junction (NMJ) area measurements were made using MetaMorph software. For gem number counts, fixed primary dermal fibroblasts were blocked and incubated with anti-SMN antibody as described previously [31]. Images were obtained using an LSM META 510 laser-scanning confocal microscope (Carl Zeiss), and SMN nuclear localization in fibroblasts was confirmed by 4',6-diamidino-2-phenylindole (DAPI) counterstaining.

### Hepatic and renal function test

Blood samples were collected from the facial vein of the mice using a lancet. The samples were then mixed with ethylenediaminetetra-acetic acid (EDTA). The solution was then centrifuged for 30 minutes at 4,700 *g*. The serum samples from each group of mice were analyzed using a DRI-CHEM clinical chemistry analyzer (FDC 3500; FujiFilm Medical Co, Tokyo, Japan) for glutamate oxaloacetate transaminase (GOT), glutamate pyruvate transaminase (GPT), blood urea nitrogen (BUN), and creatinine (CRE).

### Determination of vanadium

To investigate vanadium levels in tissue, snap-frozen kidney and liver tissues and blood were weighed and homogenized in 3 ml HNO_3_. The lysates were heated to 200°C within 15 minutes and then maintained at 200°C for 25 minutes using the ultra-high throughput microwave digestion system (MARSXpress; CEM Corporation, Matthews, NC, USA). After cooling, vanadium levels were determined by inductively coupled plasma mass spectrometry (ICP-MS) (X-Series II; Thermo Fisher Scientific Inc., Waltham, MA, USA).

### Statistical analyses

The findings were confirmed by at least three independent experiments. Data were analyzed using Prism 5 software (GraphPad Software Inc., San Diego, CA, USA). Statistical significance was determined by Student's t test or one-way analysis of variance (Tukey's test). *P *< 0.05 was considered statistically significant.

## Results

### Effect of L-AA combined with SV on SMN expression closely mimics SV alone

**Figure 1:**
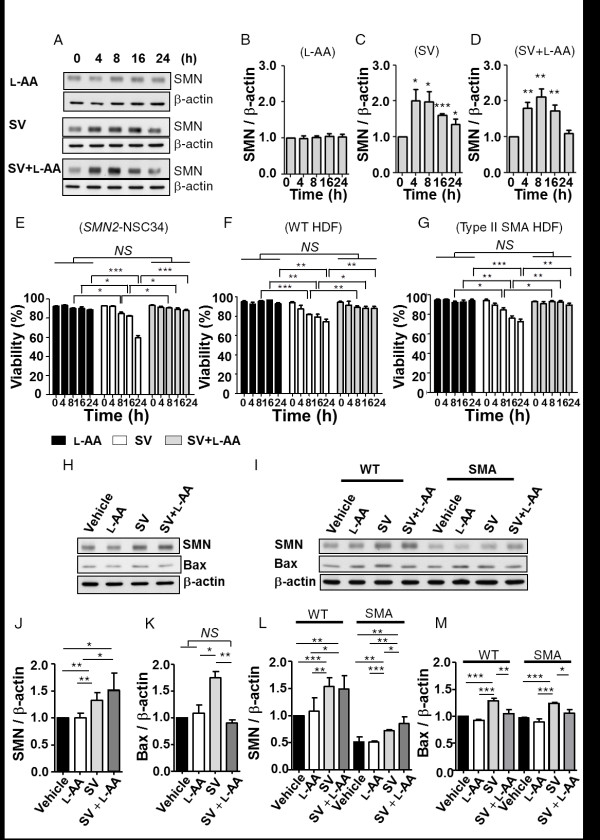
**L-Ascorbic acid (L-AA) eliminates sodium vanadate (SV)-induced cytotoxicity**. **(A) **Western blots showing survival motor neuron (SMN) expression in *SMN2*-NSC34 cells treated with 400 μM L-AA, 200 μM SV, or SV combined with L-AA at different time points. β-Actin was used as an internal control. **(B-D) **Quantification of SMN protein expression in (A). **(E-G) **Quantification of the viability of *SMN2*-NSC34 cells (E) and human dermal fibroblasts (HDFs) (F, G) treated with L-AA, SV or SV combined with L-AA. **(H, I) **Western blots showing SMN and B cell lymphoma 2-associated X protein (Bax) expression in *SMN2*-NSC34 cells (H) and HDFs (I) treated with vehicle, L-AA, SV or SV combined with L-AA. β-Actin was used as an internal control. **(J, K) **Quantification of SMN and Bax expression in (H). **(L, M) **Quantification of SMN and Bax expression in (I). The experiment was repeated at least three times, and the mean ± SEM was calculated. Statistical comparisons were performed by one-way analysis of variance (ANOVA) (E-G) and Student's t test (B-D, and J-M).*P < 0.05, **P < 0.01, and ***P < 0.001.

To examine whether L-AA disrupts the efficacy of SV, SMN protein levels in *SMN2*-NSC34 cells [31] were evaluated after treatment with SV (200 μM), SV (200 μM) combined with L-AA (400 μM), and L-AA alone (400 μM). Western blotting confirmed that L-AA treatment alone had no effect on SMN protein levels (Figure 1A, upper panel, and B). SV treatment (Figure 1A, middle panel, and C) as well as combined treatment (Figure 1A, lower panel, and D) effectively increased SMN levels from 4 to 16 h (n = 3; Figure 1A). While no statistically significant change was observed in the cells receiving combined treatment at the 24 h time point, it is possible that L-AA may accelerate the metabolism of SV, and thus reduce the drug's stability.

To extend these results to a clinically relevant model, we isolated HDFs from a type II SMA patient. The expression level of SMN protein in fibroblasts obtained from an SMA patient was approximately 59.2% of the amount observed in cells from a healthy donor (Additional file 1). The SMN protein level was restored by SV alone or combined treatment (n = 3, Figure 1I, upper panel, and L) and no change in SMN expression was observed following L-AA treatment alone in the fibroblasts derived from the SMA or healthy donors. These results demonstrate that *SMN2*-NSC34 cells receiving L-AA in addition to SV had a similar pattern of SMN-production compared to cells receiving only SV. In fibroblasts derived from healthy and SMA donors, both combined treatment and SV treatment alone resulted in increases of SMN expression. Therefore, L-AA has minimal to no negative impact on the efficacy of SV induction of SMN protein expression.

### L-AA protects cells against SV-induced cytotoxicity

SV treatment-induced cytotoxicity has been well documented [54,55]. To determine whether L-AA protects cells against SV-induced cell death, we assessed the viability of *SMN2*-NSC34 cells as well as WT and SMA patient-derived HDFs following treatment with SV (200 μM) in combination with L-AA (400 μM). We further determined whether L-AA protects *SMN2*-NSC34 cells and WT and SMA HDFs against SV-induced death by trypan blue exclusion staining (Figure 1E-G). The viability of SV-treated cells significantly decreased from 8 to 24 h in all cell types (n = 3 in each group) (Figure 1E-G, middle; Additional files 2, 3 and 4). By contrast, both NSC34 cells and HDFs that received combined treatment displayed no obvious signs of cell death from 8 to 24 h and exhibited improved viability compared with SV-treated cells as measured by trypan blue staining (Figure 1E-G, right; Additional files 2, 3 and 4). L-AA alone did not affect cell viability (Figure 1E-G, left). In addition, we found that SV-treatment resulted in increased proapoptotic Bax expression compared to L-AA treatment in *SMN2*-NSC34 cells (1.75 ± 0.11 vs 1.06 ± 0.16, *P *< 0.05), WT HDFs (1.29 ± 0.02 vs 0.92 ± 0.01, *P *< 0.001), and SMA HDFs (1.23 ± 0.01 vs 0.90 ± 0.02, *P *< 0.001) (Figure 1H, I, K, M). In contrast, combined treatment resulted in significantly lower Bax expression compared to SV treatment in *SMN2*-NSC34 cells (0.90 ± 0.06 vs 1.75 ± 0.11, *P *< 0.01), WT HDFs (1.05 ± 0.02 vs 1.29 ± 0.02, *P *< 0.01) and SMA HDFs (1.08 ± 0.04 vs 1.23 ± 0.01, *P *< 0.05) (Figure 1H, I, K, M). These results demonstrate that L-AA protected both NSC34 cells and HDFs against SV-induced toxicity.

**Figure 2:**
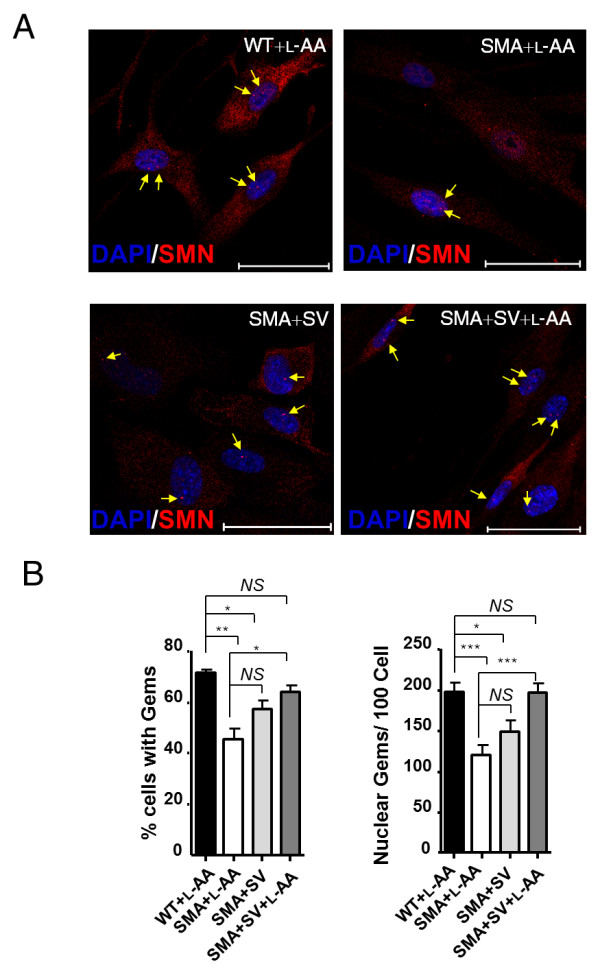
**Combined treatment restores survival motor neuron (SMN) nuclear body gems in fibroblasts of a spinal muscular atrophy (SMA) patient**. **(A) **Immunocytochemical analysis of the nuclear SMN body gems in wild-type (WT) and SMA human dermal fibroblasts (HDFs) treated with L-ascorbic acid (L-AA), sodium vanadate (SV) or L-AA combined with SV. SMN was stained with an SMN antibody (red, indicated by arrows). 4',6-Diamidino-2-phenylindole (DAPI) was used for nuclei staining. Bar: 50 μm. **(B) **The percentage of nuclei with gems (left) and the number of gems per 100 nuclei (right) in WT and SMA cells after different treatments were evaluated by immunocytochemical analysis. The mean ± SEM was calculated. *P < 0.01, **P < 0.01, and ***P < 0.001, Student's t test. NS = not significant.

Further, the degree of functional SMN recovery was determined by staining nuclear Gemini bodies/gems in WT and SMA HDFs following SV and L-AA treatment (Figure 2A, indicated by arrows). In the nucleus, SMN accumulates in nuclear bodies called 'Gems' (gemini of coiled bodies). They frequently overlap with Cajal bodies, which contain many factors involved in transcription and processing of nuclear RNAs [56]. Importantly, although their function remains unclear, gem number has been shown to correlate with disease severity, with type I SMA patients exhibiting few or no gems [57]. Fewer SMA HDFs contained nuclear gems than WT HDFs (45 ± 4% vs 71 ± 1%; *P *< 0.01; Figure 2B, left). After combined treatment, the number of SMA HDFs containing nuclear gems was significantly increased (64 ± 3%, *P *< 0.05 compared with L-AA-treated SMA HDF; Figure 2B, left). In addition, SMA HDFs treated with L-AA had fewer nuclear gems per cell than L-AA-treated WT HDFs (right; 120 ± 13 gems/100 nuclei vs 197 ± 12 gems/100 nuclei, *P *< 0.001; Figure 2B). By contrast, SV-treated SMA HDFs had an increased number of gems, although the values did not reach statistical significance (148 ± 14 gems/100 nuclei; Figure 2B, right). Of note, SMA HDFs subjected to combined treatment exhibited more gems than SV-treated SMA HDFs (195 ± 13 gems/100 nuclei vs 148 ± 14 gems/100 nuclei, *P *< 0.001; Figure 2B, right). These results indicate that combined treatment exhibits greater *in vitro *therapeutic efficacy than SV treatment alone.

### L-AA eliminates SV-induced toxicity *in vivo*

**Figure 3:**
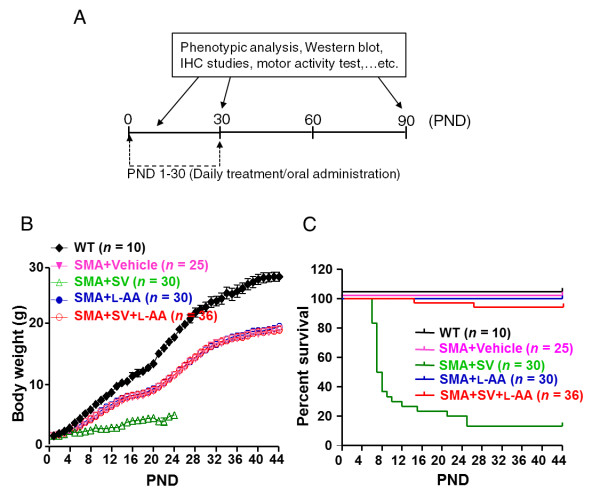
**L-Ascorbic acid (L-AA) protects against sodium vanadate (SV)-induced toxicity in a mouse model of late-onset spinal muscular atrophy (SMA)**. **(A) **Schematic indicating the treatment regimens used in this study. **(B) **The mean body weight progression of the wild-type (WT) mice (n = 10, black squares) and the SMA mice that received vehicle (n = 25, pink solid triangles), SV (n = 30, green open triangles), L-AA (n = 30, blue solid circles) and SV combined with L-AA (n = 36, red open circles) treatment. Note that SV treatment reduced the rate of body weight gain. **(C) **Survival analysis of mice that received different treatments. Note that by postnatal day (PND) 25, most of the mice that had received daily SV administration had died.

To investigate whether combined treatment has beneficial effects *in vivo*, L-AA and SV were orally administered alone or in combination from PNDs 1 to 30 to mice with late-onset SMA, which have been used previously [26,58] as a preclinical therapeutic testing system for SMA (Figure 3A). The therapeutic timeframe (PNDs 1 to 30) was designed based on several studies showing that early intervention (before PND 5) can target neurons in sufficient numbers to confer a lifespan extension [59-61]. Additionally, systemic administration of antisense oligonucleotides (ASO) targeting an *SMN2 *intronic splicing silencer on PND 1 dramatically prolongs the lifespan of SMA mice, even though inclusion of exon 7 significantly decreased after PND 30 [28], suggesting that transiently increasing SMN protein levels during the first few weeks has beneficial effects on long-term survival of SMA mice. Further, temporal restoration of SMN levels from birth to PND 28 in SMA mice with inducible SMN expression resulted in no phenotype or abnormal NMJs [62]. Therefore, the drugs were administered from PNDs 1 to 30. Our dose-response studies showed that an SV dosage of 30 mg/kg, but not 15 mg/kg, combined with L-AA causes lethality in mice (Additional file 5); therefore, a dosage of 20 mg/kg SV was selected for this study. Body weight was evaluated daily from PND 1 to PND 44, and the data revealed that SV treatment significantly slowed body weight gain (n = 30; Figure 3B). By PND 5, animals that received SV treatment were significantly underweight (2.08 ± 0.15 g) compared with those that received L-AA treatment (2.69 ± 0.10 g, n = 30), combined treatment (2.72 ± 0.10 g, n = 36), and vehicle treatment (2.64 ± 0.08 g, n = 25, *P *< 0.001; Figure 3B). It is notable that mice that received combined treatment did not exhibit the dramatically reduced weight gain seen in mice treated with SV alone (Figure 3B). In addition, 15 of 30 mice administered SV died before PND 6 (Figure 3C). The mice that received combined treatment had a survival rate similar to that of the mice that received vehicle or L-AA treatment (Figure 3C). These results provide further evidence that L-AA eliminates SV-induced toxicity in the mice with late-onset SMA.

### Combined treatment delays disease progression in mice with late-onset SMA

**Figure 4:**
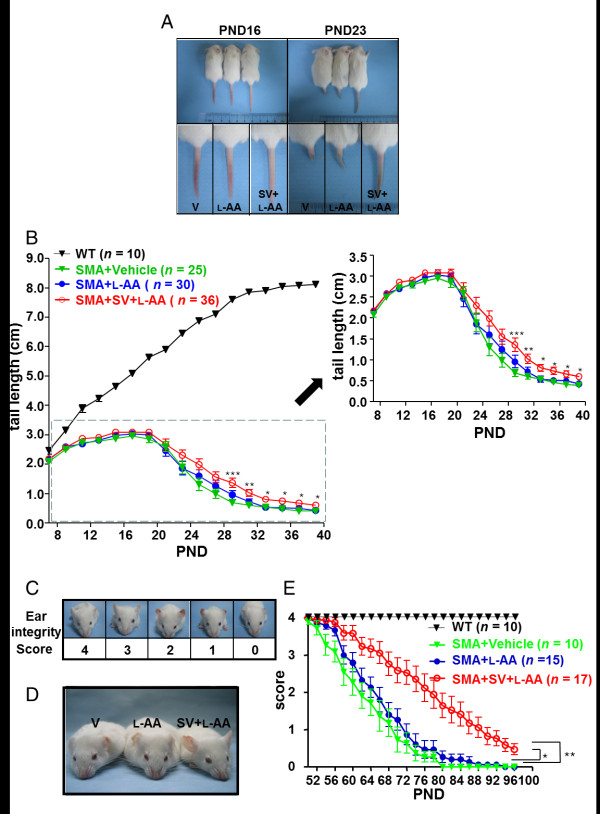
**Combined treatment delays the onset of spinal muscular atrophy (SMA) phenotype**. **(A) **Images of the mice that received different treatments on postnatal days (PNDs) 16 and 23. Note that mice that received combined treatment had longer tails. **(B) **Plot showing the tail lengths of wild-type mice (n = 10, black triangles) and mice with late-onset SMA that received vehicle (n = 25, green triangles), L-ascorbic acid (L-AA) (n = 30, blue circles) or combined (n = 36, red circles) treatment. The SMA groups are shown in a magnified figure to the right. Lengths were measured every other day from PND 7 to PND 39. A trend of delayed onset of tail necrosis was observed in the mice that received combined treatment. The combined treatment significantly delayed tail loss according to one-way analysis of variance (ANOVA). **(C) **Ear integrity was scored on a scale of 0 to 4. **(D) **PND 90 mice that had received different treatments. Note that the mice that received combined treatment retained ear integrity longer. **(E) **The plot represents the mean score of ear integrity for wild-type mice (n = 10, black triangles) and mice with late-onset SMA mice that received vehicle (n = 10, green solid triangles), L-AA (n = 15, blue solid circles) or combined (n = 17, red open circles) treatment. *P < 0.05 compared with the L-AA-treated group; **P < 0.01 compared with the vehicle-treated group, one-way ANOVA.

Distal necrosis observed in patients with SMA has also been identified in the mouse model of late-onset SMA [16,63,64]. Tail and ear necrosis have been shown to correlate with disease severity in mice with late-onset SMA, making them good indicators of the efficacy of therapeutic candidate compounds [27]. The tail length and ear integrity of mice from different treatment groups were measured every other day from PND 7 to PND 39 and from PND 50 to PND 96, respectively. The result illustrated that the SMA mice that received vehicle and L-AA treatment developed tail necrosis between PND 17 and PND 19, whereas combined treatment delayed tail necrosis until PNDs 19 to 21 (Figure 4A, B). The tails of mice that received vehicle and L-AA treatment were completely lost between PNDs 29 and 33, whereas those of mice that received combined treatment were completely lost between PNDs 37 and 39 (Figure 4B). The mice that received combined treatment displayed a significant delay (n = 30) in tail loss compared with those that received vehicle (n = 25) or L-AA (n = 36) treatment (Figure 4B). Furthermore, to characterize the degree of ear necrosis, five scores were given during each pathogenic stage (Figure 4C). The vehicle-treated (n = 10) and L-AA-treated mice (n = 15) developed ear necrosis along the edge of the ear at approximately PND 56.55 ± 1.55 and PND 59.20 ± 1.47, respectively. Of note, the mice that received combined treatment (n = 17) exhibited a significant delay in the development of ear necrosis until PND 68.00 ± 2.54 (Figure 4D, E). Approximately 11.8% (2/17) of mice with late-onset SMA that received combined treatment retained almost complete ear pinna throughout the course of measurement (Figure 4D, mice are shown on PND 90). These data indicate that combined treatment effectively delayed disease progression in the mouse model of late-onset SMA.

### Early combined treatment improves motor function in mice with late-onset SMA into adulthood

**Figure 5:**
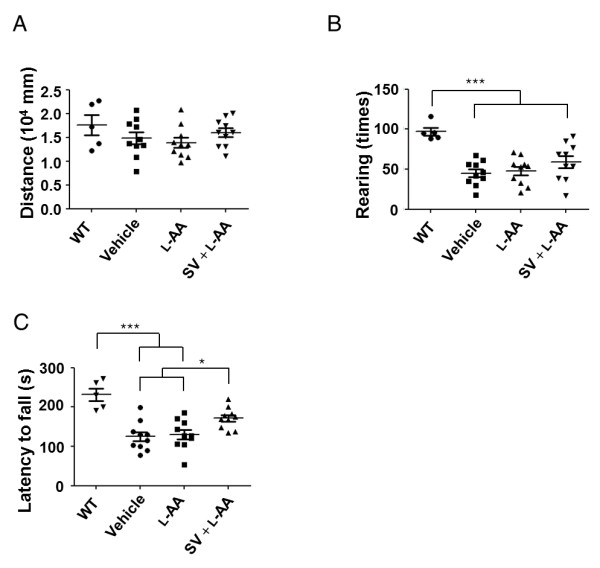
**Combined treatment improves motor function in adult mice with late-onset spinal muscular atrophy (SMA)**. **(A) **Distance traveled in the open-field test during a 60-minute period. No significant differences were observed among the treatment groups. **(B) **The frequency of rearing events in the first 10 minutes of the 60-minute experiment to examine this behavior independent of habituation. Mice that received combined treatment generally exhibited more rearing events, but this difference did not reach significance. **(C) **Average latency to fall from the rotarod for mice in response to different treatments. *P < 0.05, one-way analysis of variance (ANOVA). In all motor function tests, each circle, square or triangle represents an individual adult mouse (postnatal day (PND) 90).

As early drug administration has been demonstrated to benefit SMA therapy, whether combined treatment has beneficial effects on the motor activity of mice with late-onset SMA was further investigated. The motor functions of young mice were evaluated from PND 2 to PND 12 by the surface righting assay, tube test, and negative geotaxis assay. The different groups of mice exhibited no significant differences in the tested parameters, indicating that mice with late-onset SMA have normal motor functions at earlier developmental stages (Additional file 6). The motor functions of the adult (PND 90) mice that received treatment between PNDs 1 and 30 were further evaluated by the open-field and rotarod tests. The open-field test revealed no significant differences in the total distance traveled, indicating no difference in general activity or willingness to explore among the groups (Figure 5A). In addition, rearing events, which occur less frequently in the SMA mouse model [3,50], occurred more frequently in the mice that received combined treatment (combined treatment: 59.20 ± 7.40 events/10 minutes, n = 10; vehicle: 44.90 ± 4.79 events/10 minutes, n = 10; L-AA: 47.90 ± 5.41 events/10 minutes, n = 10), although the differences did not reach statistical significance (Figure 5B). The locomotor activities of each group were also analyzed by the accelerating rotarod test. The mice that received combined treatment stayed on the rotating cylinder longer than those that received vehicle or L-AA treatment (combined treatment: 171.30 ± 8.47 s; vehicle: 124.90 ± 11.56 s; L-AA: 129.80 ± 12.02 s; n = 10 in each group, *P *< 0.05; Figure 5C). These results indicate that the mice that received combined treatment had improved motor performance. Taken together, these results indicate that combined treatment at early developmental stages is sufficient to improve the motor activity of mice with late-onset SMA in adulthood.

### Combined treatment displays motor neuron protective effects and reduces muscle atrophy in mice with late-onset SMA

**Figure 6:**
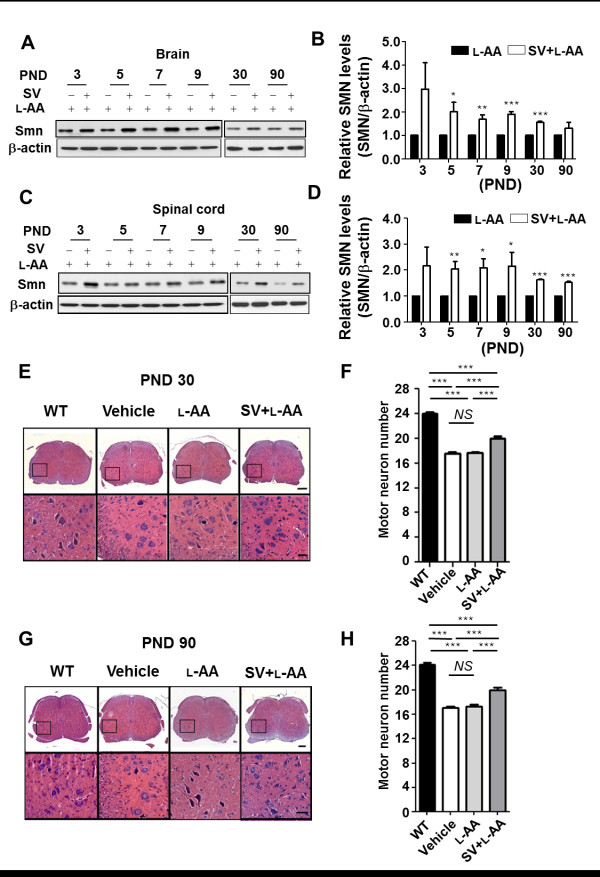
**Combined treatment increases survival motor neuron (SMN) expression and motor neuron numbers in mice with late-onset spinal muscular atrophy (SMA)**. **(A, C) **Western blots showing SMN expression in the brain (A) and spinal cord (C) in mice with late-onset SMA that received L-ascorbic acid (L-AA) or combined (n = 4 in each group) treatment. β-Actin was used as an internal control. **(B, D) **Quantification of SMN protein expression in (A, C). The mean ± SEM was calculated. **(E, G) **Histological staining of lumbar spinal cord samples on postnatal days (PNDs) 30 (E) and 90 (G) for the wild-type (WT) mice and mice with late-onset SMA that had received different treatments. Scale bar: 100 μm (E and G, upper panel); 50 μm (E and G, lower panel). **(F, H) **Quantification of motor neuron numbers in the spinal cords obtained from (E) and (G) (n = 3, 40 sections for each group were quantified). The mean ± SEM was calculated. ***P < 0.001, Student's t test.

The improved motor performance in the adult mice led us to further assess the potential therapeutic effects underlying combined treatment during development. Spinal cord and brain tissue samples were obtained at PNDs 3, 5, 7, 9 and 30 days from mice treated from PND 1 as well as from adult mice (PND 90) that received treatment from birth through PND 30. SMN expression was then assayed by western blotting. The results demonstrated that the SMA mice that received combined treatment had relative increases in SMN levels in the brain and spinal cord (n = 4 in both groups) compared with the mice that received L-AA treatment (Figure 6A-D). Pathological studies were then conducted to further investigate the therapeutic effects of combined treatment in mice. The motor neuron numbers in the WT mice and mice with late-onset SMA that received treatment for 1 month were evaluated by histology (Figure 6E-H). The result indicated that the young mice with late-onset SMA that received combined treatment for 1 month had significantly higher motor neuron numbers than those that received L-AA or vehicle treatment (19.95 ± 0.34 vs 17.65 ± 0.20 and 17.55 ± 0.19, *P *< 0.001; Figure 6F). Moreover, the adult mice with late-onset SMA that received combined treatment exhibited higher motor neuron numbers than those that received L-AA or vehicle treatment (20.00 ± 0.34 vs 17.33 ± 0.28 and 17.10 ± 0.23, *P *< 0.001; Figure 6H).

**Figure 7:**
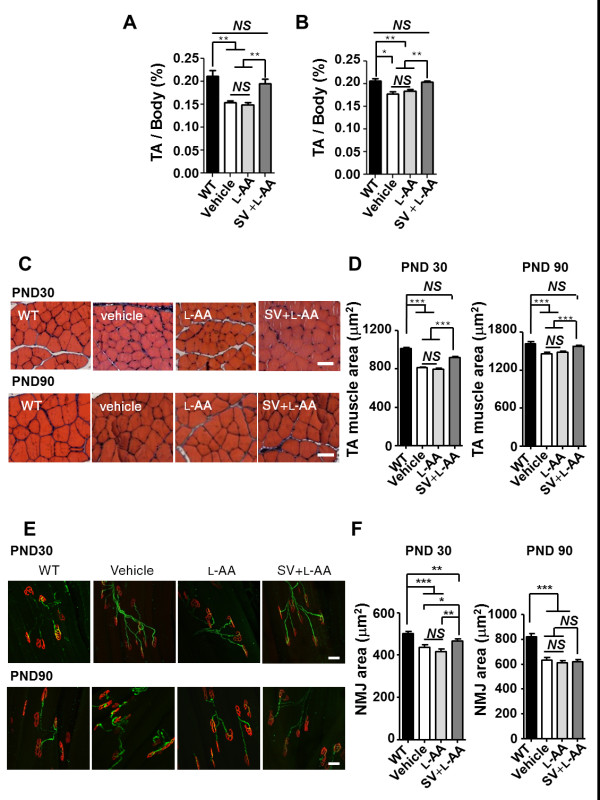
**Combined treatment improves muscle pathology in mice with late-onset spinal muscular atrophy (SMA)**. **(A, B) **The tibialis anterior (TA) muscle weight-to-body weight ratio in mice with late-onset SMA in response to different treatments on postnatal days (PNDs) 30 and 90 (n = 3 in each group). The mean ± SEM was calculated. **P < 0.01 and ***P < 0.001, Student's t test. NS = not significant. **(C) **Histological assessment of hematoxylin and eosin (H&E)-stained TA muscle from PND 30 (upper panel) and 90 (lower panel) wild-type (WT) mice or mice with late-onset SMA that received different treatments. Scale bar: 50 μm. **(D) **Quantification of the muscle area (μm^2^) in the PND 30 (left) and 90 (right) mice (n = 3, >500 myofibers for each group were quantified) obtained in (C). The mean ± SEM was calculated. **P < 0.01 and ***P < 0.001, Student's t test. NS = not significant. **(E) **Staining with the axonal marker neurofilament H (green) and neuromuscular junction (NMJ) marker α-bungarotoxin (α-BTX) (red) revealed NMJs in the treated PND 30 and 90 WT or SMA mice (n = 3). Bar: 50 μm. **(F) **Quantification of the NMJ area (μm^2^) in the PND 30 (left) and 90 (right) mice in each group obtained in (E). The mean ± SEM was calculated. **P < 0.01 and ***P < 0.001, Student's t test. NS = not significant.

Additionally, TA muscles were dissected from the WT mice and mice with late-onset SMA on PNDs 30 and 90, and the TA muscle mass-to-body weight ratio, TA muscle area, and NMJ area in TA muscle were determined. The result revealed that the PND 30 and 90 mice with late-onset SMA that received vehicle or L-AA treatment had significantly lower TA muscle-to-body weight ratios than the WT mice (n = 3 in each group, *P *< 0.01; Figure 7A, B). The TA muscle-to-body weight ratio was recovered in the mice with late-onset SMA that received combined treatment (*P *< 0.01; Figure 7A, B), indicating that combined treatment prevented muscle atrophy in those mice. In addition, histological studies revealed a decrease in the TA muscle area in the PND 30 and 90 mice with late-onset SMA compared with the WT mice (n = 3 in each group, *P *< 0.001; Figure 7C, D). By contrast, an improved TA muscle area was observed in the mice with late-onset SMA that received combined treatment (*P *< 0.001; Figure 7C, D). Immunohistochemical studies also revealed a significantly decreased NMJ area in the PND 30 and 90 mice with late-onset SMA compared with the WT mice (n = 3 in each group, *P *< 0.001; Figure 7E, F). The mice with late-onset SMA that received combined treatment exhibited an improvement in the NMJ area on PND 30 but displayed no significant differences on PND 90 (*P *< 0.05; Figure 7E, F). Collectively, these findings demonstrate that early and constant intervention with combined treatment for 1 month is sufficient to protect mice with late-onset SMA against motor neuron death and to reduce muscle atrophy.

### Combined treatment decreases Bax levels during development of mice with late-onset SMA

**Figure 8:**
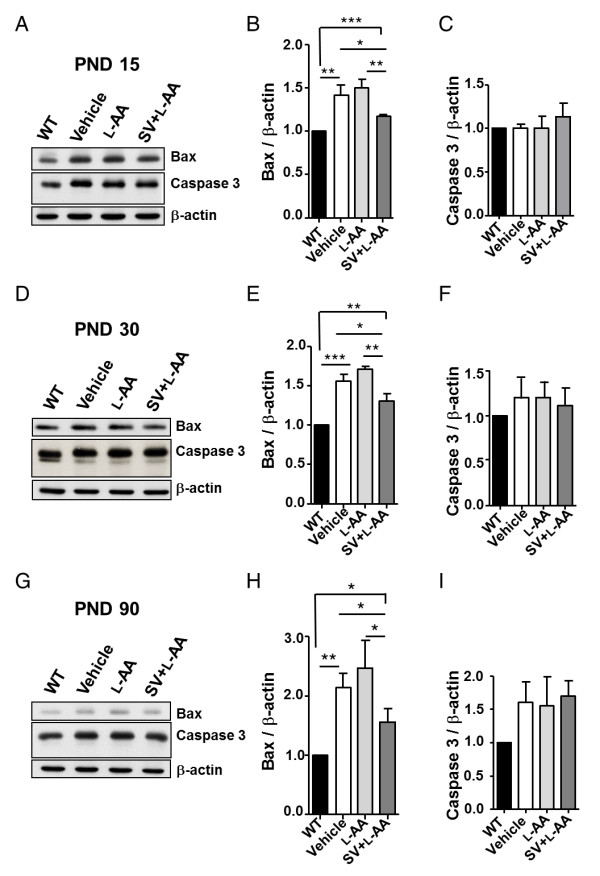
**Combined treatment decreases B-cell lymphoma 2-associated X protein (Bax) expression in the spinal cord of mice with late-onset spinal muscular atrophy (SMA)**. **(A, D, G) **Western blots showing Bax and caspase 3 expression in spinal cord samples from wild-type (WT) mice or mice with late-onset SMA on postnatal days (PNDs) 15 (A), 30 (D) and 90 (G) that received vehicle, L-ascorbic acid (L-AA) or L-AA combined with sodium vanadate (SV) (n = 3 in each group). **(B, C, E, F, H, I) **Quantification of the western blot results in (A), (D) and (G). The mean ± SEM was calculated. *P < 0.05, **P < 0.01 and ***P < 0.001, Student's t test.

Evidence from several studies also revealed abnormal Bax expression in the SMA mouse model and indicated that abolishing Bax expression improved motor neuron survival [65]. SV also induces Bax *in vitro*; however, the combined treatment eliminates Bax upregulation (Figure 1H, I, K, M). We therefore examined Bax levels in the WT mice and mice with late-onset SMA that received vehicle, L-AA or combined treatment. The results of western blotting were consistent with those of the cell line studies, indicating significantly higher Bax levels in the mice with late-onset SMA on PNDs 15 (1.42 ± 0.07-fold, *P *< 0.01), 30 (1.56 ± 0.05-fold, *P *< 0.01), and 90 (2.14 ± 0.14-fold, *P *< 0.01) compared to the WT mice (n = 3 in each group; Figure 8A, B, D, E, G, H). However, the mice that received combined treatment had significantly lower Bax levels in their spinal cords than those that received L-AA or vehicle treatment on PNDs 15 (combined treatment: 1.17 ± 0.01; L-AA: 1.50 ± 0.06 (*P *< 0.01 vs combined treatment); vehicle: 1.42 ± 0.07 (*P *< 0.05 vs combined treatment)), 30 (combined treatment: 1.31 ± 0.05; L-AA: 1.71 ± 0.02 (*P *< 0.01 vs combined treatment); vehicle: 1.56 ± 0.05 (*P *< 0.05 vs combined treatment)), and 90 (combined treatment: 1.55 ± 0.14; L-AA: 2.46 ± 0.27 (*P *< 0.05 vs combined treatment); vehicle: 2.14 ± 0.14 (*P *< 0.05 vs combined treatment)). Regardless of treatment, the mice with late-onset SMA exhibited higher Bax levels (1.17-fold to 2.46-fold) than the WT mice (n = 3 in each group; Figure 8A, B, D, E, G, H). These results indicate that combined treatment may protect motor neuron cells from death caused by decreased Bax expression *in vivo*. We also examined caspase 3 expression on PNDs 15, 30, and 90; however, no significant differences were observed (Figure 8A, C, D, F, G, I).

### Combined treatment does not affect hepatic and renal functions in mice with late-onset SMA

Toxicity in the liver and kidneys is the principal concern limiting the clinical application of vanadium [32,38]. To evaluate the safety, feasibility, and practicality of combined therapy for SMA treatment (PNDs 1 to 30), the hepatic and renal functions of SMA mice that received L-AA alone and in combination with SV were examined on PNDs 30 and 90. The results revealed no dramatic differences in the levels of GOT or GPT, which reflect hepatic function, or BUN or CRE, which reflect renal function, between the WT mice and mice with late-onset SMA that received treatment (Table 1). Importantly, all values in the mice with late-onset SMA that received combined treatment were within the reference range, indicating that the combined treatment causes no obvious organ toxicity. In addition, vanadium accumulation in the liver and kidneys was determined on PNDs 6, 30 and 90. The mice with late-onset SMA that received SV alone exhibited vanadium accumulation in the liver (2.11 ± 0.18 μg/g) and kidneys (3.89 ± 0.60 μg/g) on PND 6 (Table 2), and most of them died before PND 30 (Figure 3C). However, combined treatment significantly reduced vanadium accumulation in the liver (0.59 ± 0.01 μg/g, n = 3) and kidneys (1.37 ± 0.26 μg/g, n = 3) compared with SV treatment on PND 6. Further, vanadium accumulation in the adult mice that received combined treatment declined to very low levels by PND 90 (liver: 0.08 ± 0.04 μg/g, n = 3; kidneys: 0.07 ± 0.02 μg/g, n = 3; Table 2). Taken together, these data indicate that although vanadium is still detectable in the organs of SMA mice that receive combined treatment, L-AA limits its accumulation and preserves hepatic and renal function.

**Table 1:** Hepatic and renal function in wild-type (WT) mice and vehicle-treated, sodium vanadate (SV)-treated, L-ascorbic acid (L-AA)-treated, and L-AA+SV-treated spinal muscular atrophy (SMA) mice

Groups	GOT (U/I)	GPT (U/I)	BUN (mg/dl)	CRE (mg/dl)
Reference range	54 to 298	17 to 77	8 to 33	0.2 to 0.9
Postnatal day 30				
WT	55.67 ± 10.20	35.67 ± 6.67	19.82 ± 0.56	< 0.20
SMA (vehicle)	93.69 ± 20.28	30.16 ± 2.64	22.22 ± 0.77	0.23 ± 0.02
SMA (L-AA)	129.62 ± 33.65	31.15 ± 2.14	23.98 ± 0.91	0.26 ± 0.02
SMA (L-AA+SV)	119.87 ± 28.25	31.27 ± 2.99	22.80 ± 1.01	0.21 ± 0.03
Postnatal day 90				
WT	125.40 ± 30.09	57.60 ± 7.63	24.38 ± 2.65	< 0.20
SMA (vehicle)	107.05 ± 18.45	42.20 ± 4.19	22.27 ± 0.90	< 0.20
SMA (L-AA)	169.54 ± 19.09	37.69 ± 2.96	24.32 ± 0.65	< 0.20
SMA (L-AA+SV)	143.50 ± 20.66	39.62 ± 3.85	25.17 ± 1.30	< 0.20

Results were determined in at least five mice in each group, and the mean ± SEM was calculated. BUN = blood urea nitrogen; CRE = creatinine; GOT = glutamate oxaloacetate transaminase; GPT = glutamate pyruvate transaminase.

**Table 2:** Accumulation of vanadium (μg/g) in the liver, kidneys, and blood of vehicle-treated, sodium vanadate (SV)-treated, L-ascorbic acid (L-AA)-treated, and L-AA+SV-treated spinal muscular atrophy (SMA) mice

Groups	Liver	Kidney	Blood
Postnatal day 6:			
SMA (vehicle)	ND	ND	
SMA (SV)	2.11 ± 0.18	3.89 ± 0.60	
SMA (L-AA)	ND	ND	
SMA (L-AA+SV)	0.59 ± 0.01 ***	1.37 ± 0.26 *	
Postnatal day 30:			
SMA (vehicle)	ND	ND	ND
SMA (L-AA)	ND	ND	ND
SMA (L-AA+SV)	0.22 ± 0.02	0.22 ± 0.04	0.07 ± 0.01
Postnatal day 90:			
SMA (vehicle)	ND	ND	ND
SMA (L-AA)	ND	ND	ND
SMA (L-AA+SV)	0.08 ± 0.04	0.07 ± 0.02	0.02 ± 0.01

Results are reported as means of three mice in each group ± SEM. *P < 0.05 and ***P < 0.001 relative to SV. ND = not detectable (< 0.202 ng/g).

## Discussion

In the present study, we show that L-AA largely decreases vanadium toxicity both *in vitro *and *in vivo *and that administration of SV combined with L-AA delays disease progression, improves motor activities and muscle pathology, and protects spinal motor neurons in a mouse model of late-onset SMA.

### Early intervention with combined treatment provides long-term efficacy in mice with late-onset SMA

Several reports indicated that SMA mice that received treatment before disease onset exhibited a satisfactory recovery of SMN levels [17,20,22,27,28,66], an improvement in SMA symptoms [17,20,22,27,28,66], and rescue of the SMA-like phenotype [28,66]. Based on these results, SMA mice received combined treatment for 1 month beginning on PND 1. The initial results revealed improved SMN levels in the brains and spinal cords of the mice that received combined treatment relative to those of the mice that received L-AA alone (Figure 6A-D). In addition, combined treatment improved the motor performance of the adult mice with late-onset SMA (Figure 5C), most probably because of a protective effect on motor neurons (Figure 6E-H) and an improvement in muscle pathology (Figure 7) due to the recovery of SMN levels. These findings are consistent with the fact that SMN is required for normal development and that early treatment resulting in sufficient SMN levels improves the prognosis in SMA models. While early treatment has been shown to be essential to mitigating disease severity in mice with early-onset SMA, whether early drug intervention is also necessary for mice with late-onset SMA has not been established. Hua *et al*. reported that early (E15) ASO injection in mice with late-onset SMA [26] dramatically reduced the disease severity and improved motor function, indicating that early treatment is also beneficial in late-onset SMA. However, most cases of type II and III SMA are not diagnosed in the earlier stages of the disease, making it necessary to evaluate the efficacy of later interventions in the late-onset SMA model. In this study, we did not evaluate whether combined treatment administered to mice with late-onset SMA near or after the onset of symptoms could achieve ideal therapeutic effects. However, there has been some evidence that other drugs such as SV that correct the *SMN2 *alternative splicing may be therapeutic even when administered later in the disease progression. Some reports have indicated that type II SMA patients that receive VPA for 6 months at disease onset showed significant increases in muscle strength and function [67,68]. Additionally, some type II and III SMA patients who received salbutamol treatment for 6 months at disease onset also showed an increase of muscle strength [69]. These findings therefore support the possibility that combined L-AA and SV treatment applied at later stages of late-onset SMA may be beneficial and present a promising avenue for further study. However, the efficacy of later interventions with combined treatment should be further investigated. SV treatment (20 mg/kg once daily) alone caused substantially reduced weight gain and mortality before PND 6 in mice (Figure 3). Decreasing the SV dosage to 15 mg/kg prevented lethality but still resulted in a reduced growth rate in juvenile mice (Additional file 5). By contrast, the mice that received combined treatment displayed normal growth rates with no obvious hepatic or renal damage (Figure 3 and Table 1) and reduced vanadium accumulation in the liver and kidneys on PND 30 (Table 2) that decreased to very low levels in the adult mice after drug therapy ended. The effects of SV on the liver or kidneys are of particular concern because of their involvement in the excretory mechanism [43,44]. In addition, a report indicated that the daily tolerance of vanadium ranges from 10 to 60 μg/day in humans [70]. However, the average basal and normative vanadium requirement has been difficult to ascertain. Data acquired from deprivation studies in animals indicated that 2 to 25 ng/day vanadium often induced no significant clinical effects, and many diets supply approximately 15 to 30 μg of vanadium daily, suggesting that dietary intake of vanadium of approximately 10 μg/day is safe [71]. Moreover, the addition of L-AA dramatically reduces SV-induced toxicity *in vitro *and *in vivo *(Figures 1 and 3). L-AA has very low toxicity, and the minimum dietary requirement in humans is generally 40 to 100 mg/day, however, concentrations of up to 100-fold higher have been shown to be within a safe range [72]. Therefore, combined treatment provides a novel and useful strategy for SMA therapy in the near future.

### Combined treatment results in reduced Bax levels and attenuated motor neuron death

Motor neuron loss has been found in the lumbar spinal cord in all types of SMA [73]. Inhibition of neuronal apoptosis is one potential strategy for SMA therapy [18,58,65]. The proapoptotic protein Bax is involved in neuron death after trophic factor deprivation and during development [74] and is induced by SV treatment [54,55]. Abolishing Bax-dependent apoptosis prolongs lifespan in a mouse model of type I SMA [65], indicating that Bax may play a deleterious role in SMA pathogenesis. Although SV enhances *SMN2 *expression [31], the toxic effect of SV on cells (especially NSC34 cells and HDFs) in this study (Figure 1E-G) is a major obstacle to the application of vanadium-related compounds in SMA therapy. L-AA protects cells against SV-induced cell death (Figures 1E-G and 8). The levels of Bax, but not of caspase 3, were significantly downregulated in cells (Figure 1H, I, K, M) and animals (Figure 8) that received combined treatment, indicating that L-AA protects motor neurons from death caused by decreased Bax expression through a Bax-dependent mechanism. However, the Bax level remained higher in the mice with late-onset SMA that received combined treatment than in the WT mice (Figure 8). It is possible that L-AA only functions to eliminate SV-induced Bax levels but fails to reverse SMA pathogenesis. In addition, we attributed the reduced Bax levels observed in the mice that received combined treatment (Figure 8) to SMN induction.

### Future perspectives of combined treatment

Vanadate is a small compound that can pass through the blood-brain barrier. It moves through the circulatory system and enters the metabolic pathways [75]. The vanadate derivatives bis(ethylmaltolato)oxovanadium(IV) and bis(maltolato)oxovanadium(IV) are insulin-mimetic agents currently being investigated in phase II clinical trials for type II diabetes [35]. Those drugs may present novel opportunities for SMA therapy in the near future. Although some reports indicated that vanadate is not toxic when administered orally [32], the effects of vanadium accumulation in organs and the toxic effects of long-term administration of vanadate-based compounds need to be carefully investigated. Also, the optimal timing of combined treatment (that is, when to begin and the duration), in addition to the optimal time for additional courses of drug administration, also need to be established. Furthermore, L-AA appears to be an ideal chelating agent to combine with vanadium compounds. However, other chelating agents that are effective in combination with vanadium compounds are being investigated in a diabetic model. The ingestion of a tea decoction with vanadium results in reduced vanadium accumulation in most tissues [76], and when administered orally over 14 months, this combination induces long-term glycemic stability without obvious organ toxicity [77]. The development of improved chelating agents with strong antioxidant properties that are readily biodegradable, cost effective, and stable within a wide pH range would boost the safety and efficacy of vanadium for SMA treatment.

## Conclusions

Our work demonstrates that early treatment with vanadate combined with L-AA has considerable potential for treating patients with late-onset type II/III SMA. Furthermore, the development of a vanadate derivative and the usage of vanadium compounds in combination with chelating agents are other feasible strategies for SMA therapy.

## Abbreviations

α-BTX: α-bungarotoxin; ASO: antisense oligonucleotides; BUN: blood urea nitrogen; CRE: creatinine; GOT: glutamate oxaloacetate transaminase; GPT: glutamate pyruvate transaminase; HDF: human dermal fibroblasts; L-AA: L-ascorbic acid; NMJ: neuromuscular junction; PND: postnatal day; SMA: spinal muscular atrophy; SMN: survival motor neuron; SV: sodium vanadate; TA: tibialis anterior; VPA: valproic acid; WT: wild-type.

## Competing interests

The authors declare that they have no competing interests

## Authors' contributions

H-CL and C-HT designed and performed experiments and wrote the manuscript. L-KT generated the HDFs from type II SMA patient and analyzed the SMN protein level in HDFs. H-CL performed the histopathological characterization of spinal cord. C-HT, H-MH-L, HL and SL-C organized this project and finalized the manuscript. All authors have read and approved the final manuscript.

## Pre-publication history

The pre-publication history for this paper can be accessed here:

http://www.biomedcentral.com/1741-7015/11/38/prepub

## Supplementary Material

Additional file 1 **Type II spinal muscular atrophy (SMA) patient-derived human dermal fibroblasts (HDFs) exhibit decreased survival motor neuron (SMN) levels**. **(A) **Western blot analysis of HDF samples from a wild-type (WT) and SMA patient. β-Actin was used as an internal control. **(B) **Quantitation of the western blot results in (A). At least three independent experiments were carried out and the mean ± SEM was calculated. ***P < 0.001, t test. Click here for file

Additional file 2 **L-Ascorbic acid (L-AA) reduces sodium vanadate (SV)-induced *SMN2*-NSC34 cell death**. Quantification of the viability of *SMN2*-NSC34 cells (Figure 1E) treated with L-AA, SV or SV combined with L-AA. Click here for file

Additional file 3 **L-Ascorbic acid (L-AA) reduces sodium vanadate (SV)-induced wild-type human dermal fibroblasts (WT-HDF) death**. Quantification of the viability of WT-HDFs (Figure 1F) treated with L-AA, SV or SV combined with L-AA. Click here for file

Additional file 4 **L-Ascorbic acid (L-AA) reduces sodium vanadate (SV)-induced spinal muscular atrophy human dermal fibroblast (SMA-HDF) death**. Quantification of the viability of SMA-HDFs (Figure 1G) treated with L-AA, SV or SV combined with L-AA. Click here for file

Additional file 5 **Determination of the lethal dose of combined treatment in type III spinal muscular atrophy (SMA) mice**. The survival rates of mice that received L-ascorbic acid (L-AA), sodium vanadate (SV) alone and combined treatment were determined. Click here for file

Additional file 6 **Combined treatment does not improve motor function at an early age in type III spinal muscular atrophy (SMA) mice**. **(A-D) **Motor functions were determined by surface righting assay (A), tube test (B) and negative geotaxis assay (C and D). Each group of mice showed no significant difference. Click here for file
