# Supplementary figures and images for: Sodium vanadate combined with l-ascorbic acid delays disease progression, enhances motor performance, and ameliorates muscle atrophy and weakness in mice with spinal muscular atrophy
Source: BMC Med. 2013 Feb 14;11:38. doi: 10.1186/1741-7015-11-38 (PMC3682891; doi:10.1186/1741-7015-11-38)

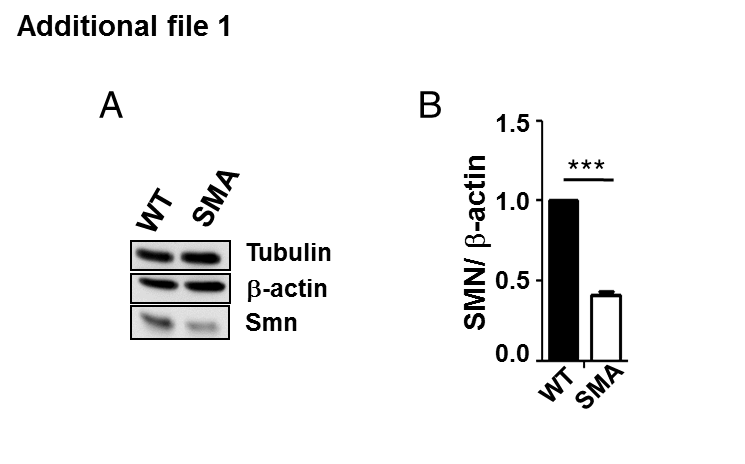

Supplement: Additional file 1 — Type II spinal muscular atrophy (SMA) patient-derived human dermal fibroblasts (HDFs) exhibit decreased survival motor neuron (SMN) levels. (A) Western blot analysis of HDF samples from a wild-type (WT) and SMA patient. β-Actin was used as an internal control. (B) Quantitation of the western blot results in (A). At least three independent experiments were carried out and the mean ± SEM was calculated. ***P < 0.001, t test. [file 1741-7015-11-38-S1.TIFF]

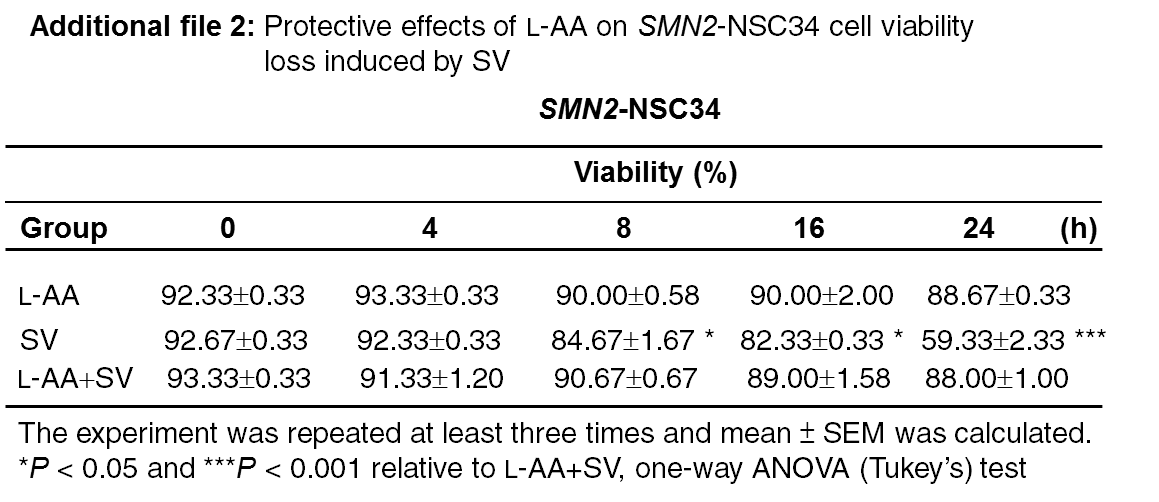

Supplement: Additional file 2 — L-Ascorbic acid (L-AA) reduces sodium vanadate (SV)-induced SMN2-NSC34 cell death. Quantification of the viability of SMN2-NSC34 cells (Figure 1E) treated with L-AA, SV or SV combined with L-AA. [file 1741-7015-11-38-S2.TIFF]

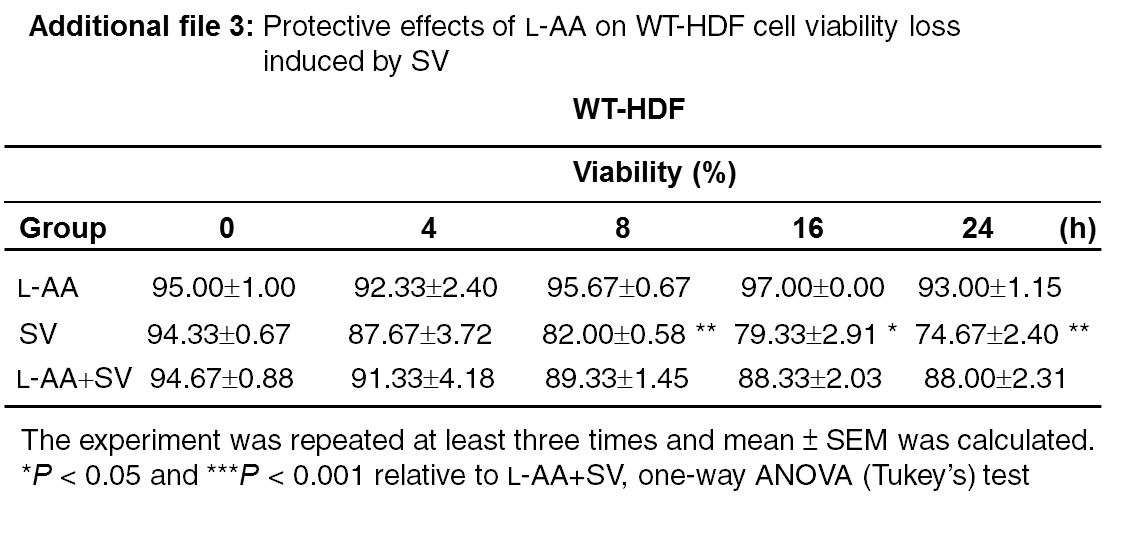

Supplement: Additional file 3 — L-Ascorbic acid (L-AA) reduces sodium vanadate (SV)-induced wild-type human dermal fibroblasts (WT-HDF) death. Quantification of the viability of WT-HDFs (Figure 1F) treated with L-AA, SV or SV combined with L-AA. [file 1741-7015-11-38-S3.TIFF]

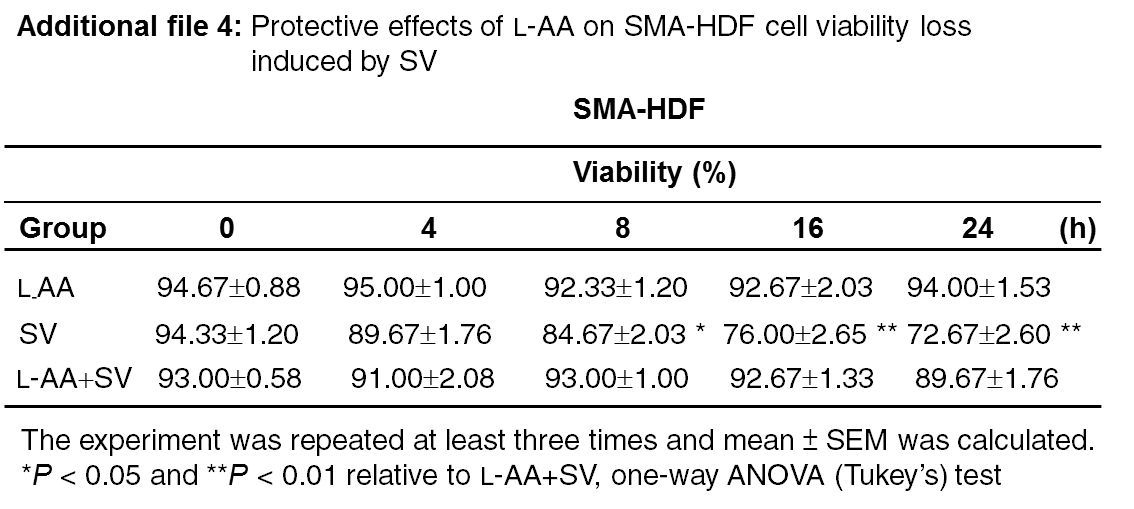

Supplement: Additional file 4 — L-Ascorbic acid (L-AA) reduces sodium vanadate (SV)-induced spinal muscular atrophy human dermal fibroblast (SMA-HDF) death. Quantification of the viability of SMA-HDFs (Figure 1G) treated with L-AA, SV or SV combined with L-AA. [file 1741-7015-11-38-S4.TIFF]

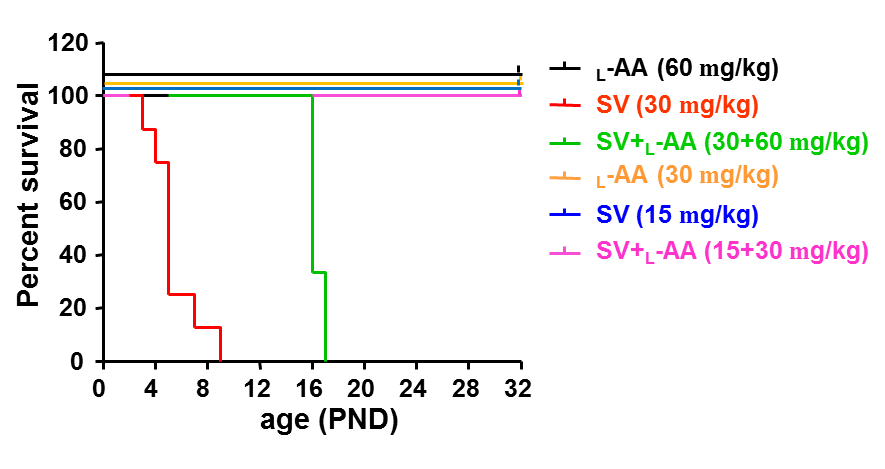

Supplement: Additional file 5 — Determination of the lethal dose of combined treatment in type III spinal muscular atrophy (SMA) mice. The survival rates of mice that received L-ascorbic acid (L-AA), sodium vanadate (SV) alone and combined treatment were determined. [file 1741-7015-11-38-S5.TIFF]

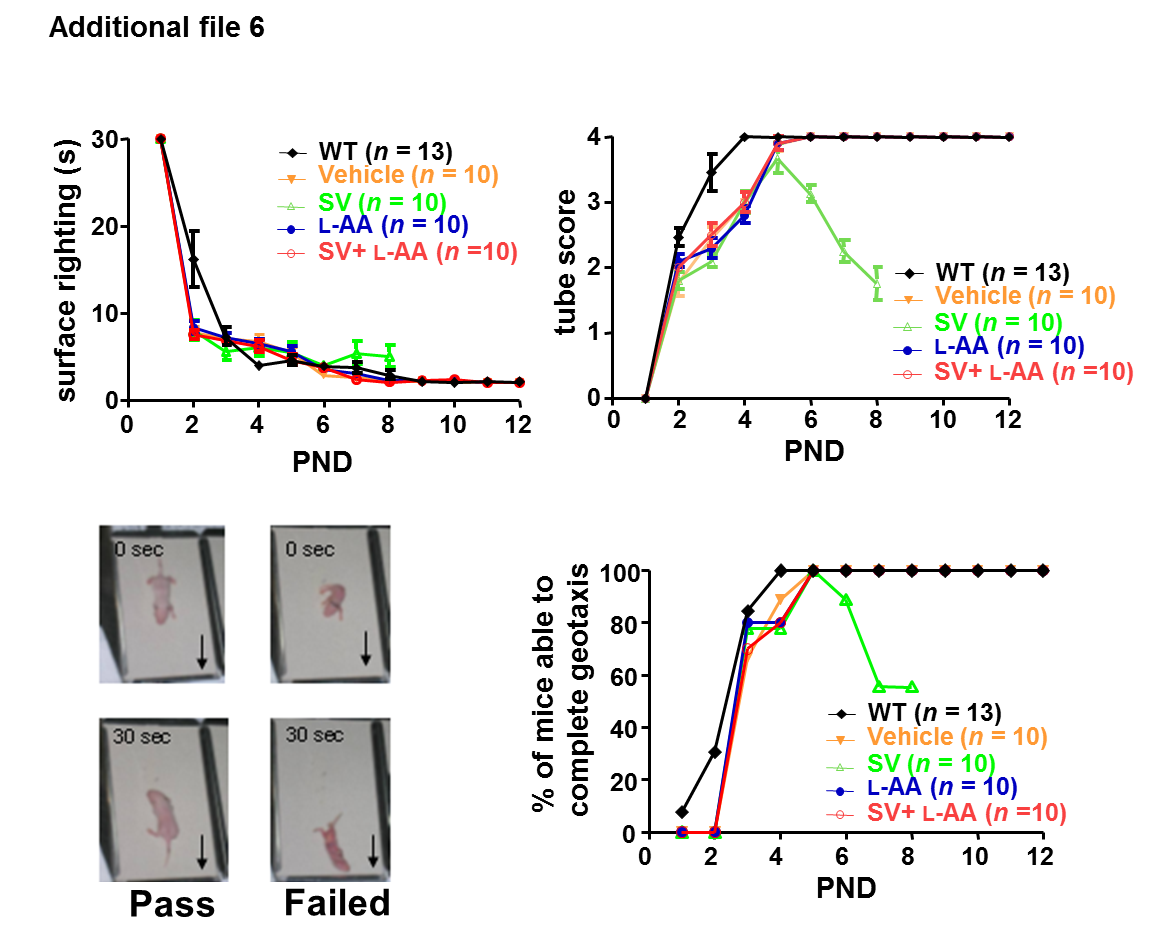

Supplement: Additional file 6 — Combined treatment does not improve motor function at an early age in type III spinal muscular atrophy (SMA) mice. (A-D) Motor functions were determined by surface righting assay (A), tube test (B) and negative geotaxis assay (C and D). Each group of mice showed no significant difference. [file 1741-7015-11-38-S6.TIFF]
